# An Aptamer for Broad Cancer Targeting and Therapy

**DOI:** 10.3390/cancers12113217

**Published:** 2020-10-31

**Authors:** Bethany Powell Gray, Xirui Song, David S. Hsu, Christina Kratschmer, Matthew Levy, Ashley P. Barry, Bruce A. Sullenger

**Affiliations:** 1 Department of Surgery, Duke Cancer Institute, Duke University School of Medicine, Durham, NC 27710, USA; bethany.gray@duke.edu (B.P.G.); xirui.song@duke.edu (X.S.); 2 Department of Medical Oncology, Duke Cancer Institute, Center for Genomics and Computational Biology, Duke University Medical Center, Durham, NC 27710, USA; shiaowen.hsu@duke.edu; 3 Department of Biochemistry, Albert Einstein College of Medicine, Bronx, NY 10461, USA or christina.kratschmer@med.einstein.yu.edu (C.K.); or matthew.levy@einstein.yu.edu (M.L.); 4 Research and Development Division, b3 bio, Inc., Durham, NC 27709, USA; ashley.barry@duke.edu

**Keywords:** aptamer, aptamer-drug conjugate, aptamer highly toxic drug conjugate, cancer, drug targeting

## Abstract

**Simple Summary:**

Recent efforts to improve chemotherapy’s antitumor effects have increasingly focused on targeted therapies, where the drug is modified with an agent able to specifically deliver it to the tumor while limiting its accumulation in normal tissue. Aptamers, comprised of short pieces of RNA or DNA, are ideal for this type of drug targeting due in part to their ease of chemical synthesis. The E3 aptamer was previously conjugated to highly toxic chemotherapeutics and shown to target and treat prostate tumors. Here, we show that E3 is not limited to prostate cancer targeting but appears to broadly target cancer cells. E3 highly toxic drug conjugates also efficiently kill a broad range of cancer types, and E3 targets tumors that closely model patient tumors. Thus, the E3 aptamer appears to be a general agent for specific delivery of chemotherapy to tumors and should improve antitumor treatment while reducing unwanted toxicities in other tissues.

**Abstract:**

Recent advances in chemotherapy treatments are increasingly targeted therapies, with the drug conjugated to an antibody able to deliver it directly to the tumor. As high-affinity chemical ligands that are much smaller in size, aptamers are ideal for this type of drug targeting. Aptamer-highly toxic drug conjugates (ApTDCs) based on the E3 aptamer, selected on prostate cancer cells, target and inhibit prostate tumor growth in vivo. Here, we observe that E3 also broadly targets numerous other cancer types, apparently representing a universal aptamer for cancer targeting. Accordingly, ApTDCs formed by conjugation of E3 to the drugs monomethyl auristatin E (MMAE) or monomethyl auristatin F (MMAF) efficiently target and kill a range of different cancer cells. Notably, this targeting extends to both patient-derived explant (PDX) cancer cell lines and tumors, with the E3 MMAE and MMAF conjugates inhibiting PDX cell growth in vitro and with the E3 aptamer targeting PDX colorectal tumors in vivo.

## 1. Introduction

The death rate among cancer patients in the United States has continually declined since 1991, reducing by almost 30% [1]. Half of this marked improvement is attributed to improvements in treatments, most notably the use of chemotherapy [2]. However, despite these advances, cancer remains the second most common cause of death among Americans, averaging more than 1,600 deaths each day [1]. Given the marked success of chemotherapy, but still the clear need for improvement, extensive efforts have focused on increasing antitumor effects while concurrently reducing the dose-limiting toxicities that result from chemotherapeutic drug accumulation in normal tissue. In an incredible realization of the vision of Paul Ehrlich, recent advances have come in the form of antibody-drug conjugates (ADCs). Over 100 years ago, Ehrlich suggested both that antibodies could be used as “magic bullets” to specifically target tumors, preventing toxicity to normal tissue, and that toxins could be attached to the antibodies to increase therapeutic potential (reviewed in [3]). Only in the past 10 years have these dreams truly become a reality, with ADCs now comprising one of the fastest-growing fields of oncologic therapeutics (reviewed in [4,5]). Between 2011 and 2018, 4 ADCs were U.S. Food and Drug Administration (FDA) approved for the treatment of cancer (reviewed in [4]), and since June of 2019 alone, 5 more ADCs gained FDA approval [6,7,8,9,10]. Currently, more than 60 different ADCs are in clinical trials (reviewed in [4,5]). While first-generation ADCs failed in the clinic (reviewed in [11]), these second-generation ADCs are succeeding due to newer, highly toxic chemotherapeutics coupled to the antibodies via more stable chemical linkers.

Although ADCs seem poised to revolutionize chemotherapy treatment, the development of ADCs is complicated, requiring antibody humanization as well as difficult chemical modifications to covalently attach the chemotherapy payload. The result is heterogenous mixtures of ADCs, with a range of drug:antibody ratios instead of a singular product. Recently we and others have shown that aptamers, small RNA or DNA ligands, can be used in place of antibodies to create a new type of targeted chemotherapeutic: aptamer highly toxic drug conjugates (ApTDCs) [12,13,14]. Unlike antibodies, aptamers (reviewed in [15,16,17]) do not require humanization and are easily amenable to chemical synthesis and modification, allowing for synthesis of a defined drug product. Additionally, aptamers obtain antibody-like affinities for their targets, functioning with antibody specificity at a fraction of the size, which allows for additional tissue penetration. 

To demonstrate the clinical potential of ApTDCs, we previously selected a new prostate-cancer specific RNA aptamer, termed E3 [12]. E3 was identified through a cell selection technique, based upon the traditional process of aptamer selection via Systematic Evolution of Ligands by EXponential enrichment (SELEX) [18,19], which involves iterative rounds of binding of a random oligonucleotide library against a cellular target instead of a traditional protein target. To specifically select for aptamers that not only target prostate cancer cells but also internalize into those cells, we used Cell-Internalization SELEX (reviewed in [20]), which excludes aptamers that bind the cell surface, isolating and identifying only those aptamers that enter the cells. Two separate Cell-Internalization SELEX assays against four different prostate cancer cell lines coupled with negative selections against normal prostate epithelial cells identified the E3 aptamer as a prostate cancer-specific ligand with specificity for cancerous versus normal prostate cells. Confocal microscopy confirmed the internalization of E3 into prostate cancer cells via active-targeting receptor-mediated endocytosis, resulting in accumulation in lysosomes.

To closely model E3-based ApTDCs after clinically successful ADCs, we chemically conjugated E3 to the highly toxic auristatin drugs monomethyl auristatin E (MMAE) and monomethyl auristatin F (MMAF), both potent tubulin inhibitors that are too toxic to be used alone but are ideal for efficacious tumor-targeted therapy [21,22]. Both the E3 MMAE and MMAF drug conjugates efficiently killed prostate cancer cells without affecting normal prostate cell viability. Importantly, the E3 aptamer maintained specificity for prostate tumors in vivo, as evidenced by near-infrared (NIR) fluorescent imaging of Alexa Fluor 750-labeled E3 localization to prostate xenografts in mice. Most notably, treatment with MMAF-E3 conjugate significantly inhibited prostate tumor growth and prolonged survival in mice, demonstrating for the first time in vivo efficacy of an ApTDC. 

In this study, we demonstrate that, despite its selection against prostate cancer cells, the E3 aptamer also targets numerous other human cancer cell types, including breast, pancreatic, lung, colorectal, cholangiocarcinoma, glioblastoma, neuroblastoma, leukemia, renal, and skin cancers. Of particular note is the ability of E3 to target patient-derived xenograft (PDX) colorectal, cholangiocarcinoma, osteosarcoma, and renal cancer cell lines, demonstrating that the aptamer is able to target clinically relevant cell lines. We also show that the E3 MMAE and MMAF highly toxic drug conjugates can be used for efficient drug targeting and drug-induced cell killing against the different types of cancer cells that E3 targets, including PDX colorectal cancer cells. Most importantly, E3 is also able to target PDX colorectal tumors in mice. 

Thus, E3 appears to serve as a generalizable cancer targeting ligand, able to specifically target every cancer type tested to date. The activity of the E3 drug conjugates against different cancer types coupled with the ability of the E3 aptamer to target PDX tumors in mice suggests that the E3 aptamer may prove useful for targeted delivery of highly toxic drugs to a wide variety of cancers in patients.

## 2. Results

### 2.1. E3 Aptamer Targeting Across Human Cancer Types

We previously demonstrated that the synthesis of fluorescent dye-labeled E3 retains aptamer function and specificity for prostate cancer cells [12]. Conjugation of a dye such as DyLight 650 (DL650) to E3 allows for easy visualization and quantification of aptamer targeting to cells via flow cytometry. To determine whether E3 targets other cancer types aside from prostate cancer, we incubated a panel of 11 different cancer cell lines with increasing concentrations of DL650-E3. This panel included three different breast cancer cell lines (MDA-MB-231, MCF7 and BT-474), three different pancreatic cancer cell lines (PANC-1, MIA PaCa-2 and BxPC-3), 2 different brain cancer lines (U-118MG and SK-N-AS), lung cancer cells (NCI-H1703), Acute Lymphoblastic Leukemia cells (Jurkat) and skin cancer cells (A431). These various cell lines were chosen to represent the heterogeneity of different subtypes of cancer (for cell classifications and defining characteristics, see Table S1). 

**Figure 1:**
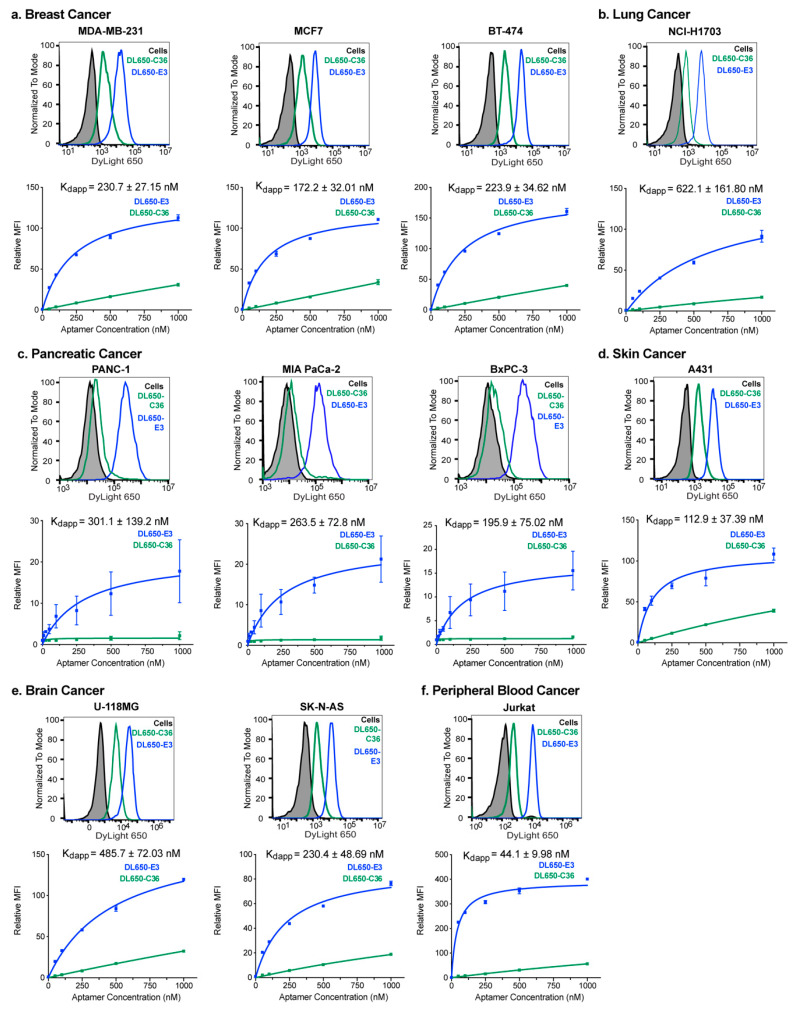
The E3 aptamer targets a broad range of cancer cell types with nanomolar-apparent affinity. Cancer cells were incubated with increasing concentrations of DL650-E3 aptamer or DL650-C36 control aptamer for 1 h before washing cells and analyzing by flow cytometry. The histograms are graphs of cell fluorescence after treatment with the 250 nM (**a**,**b**,**d**–**f**) or 1000 nM (**c**) aptamer solutions, and the binding curves are the combination of three independent experiments, with the median fluorescent intensity (MFI) of the aptamer-treated samples normalized to the median fluorescent intensity of the cells alone signal. Flow cytometry analysis of E3 targeting to (**a**) the breast cancer cell lines MDA-MB-231, MCF7 and BT-474; (**b**) the lung cancer cell line NCI-H1703; (**c**) the pancreatic cancer cell lines PANC-1, MIA PaCa-2 and BxPC-3; (**d**) the skin epidermoid cancer cell A431; (**e**) the brain cancer cell lines U-118MG and SK-N-AS; and (**f**) the Jurkat peripheral blood cancer cell line.

Interestingly, E3 demonstrated targeting of each cancer cell line tested, specifically binding the cancer cells when compared to a nonspecific size-matched control aptamer C36 (cntrl.36 [23]), which is the same length as E3 (Figure 1, Figure S1). While E3 targeting to the various cancer cells increased with increasing aptamer concentration, the control aptamer exhibited little cell staining, even at high aptamer concentrations. To further ensure that the E3 aptamer targeting to cancer cells resulted from specific targeting as opposed to nonspecific oligonucleotide binding, salmon sperm DNA was included in the incubations as a general oligonucleotide blocking agent, much as excess protein is used to block nonspecific protein uptake for antibody incubations. E3 binding affinity for each cell line was determined using nonlinear fits for the graphs of relative median fluorescence vs. aptamer concentration to give apparent dissociation constants (K_ds_).

As an approximation of some of the diversity of breast patient tumors, we tested both breast adenocarcinoma cells, MDA-MB-231 cells, and invasive ductal carcinoma cells, MCF7 and BT-474 cells. These three breast cancer cell lines were also chosen as they span the spectrum of breast cancer cell receptor classifications, representing different levels of estrogen receptor (ER), progesterone receptor (PR) and HER2 positivity. BT-474 cells are positive for all three receptors, MCF7 cells ER+, PR+ and HER2− and MDA-MB-231 cells classified as triple-negative breast cancer (ER−, PR−, HER2−) (reviewed in [24]). DL650-E3 targeted all 3 of these breast cancer subtypes with apparent binding affinities (K_d_s) ranging from 172–231 nM (Figure 1a). 

Additionally, E3 targeted the lung cancer squamous cell carcinoma line NCI-H1703. Despite demonstrating the lowest affinity for E3 of all cells tested, the NCI-H1703 cells still displayed a nanomolar affinity for the aptamer (K_d_ = 622 nM) (Figure 1b). More than 90% of all pancreatic cancer is pancreatic ductal adenocarcinoma (PDAC) which alone is the fourth most common cause of cancer deaths worldwide (reviewed in [25]). Thus, we analyzed E3 targeting to three different PDAC cell lines. DL650-E3 targeted all 3 of these PDAC cell lines with apparent binding affinities (K_d_s) ranging from 196—301 nM (Figure 1c). E3 also targeted the skin epidermoid carcinoma A431 cells with midrange affinity (K_d_ = 113 nM) (Figure 1d). 

To examine E3 targeting to different types of brain cancer, we examined E3 targeting to both glioblastoma cells (U-118MG) and neuroblastoma cells (SK-N-AS). DL650-E3 targeted both cell lines, with higher affinity binding to the SK-N-AS neuroblastoma cells (K_d_ = 230nM) compared to the U-118MG glioblastoma cells (K_d_ = 486nM) (Figure 1e). Finally, E3 targeted Acute Lymphoblastic Leukemia (ALL) Jurkat cells. The ALL cells had the highest affinity for E3 of all the cell lines tested, with a K_d_ of 44nM (Figure 1f). Significantly, E3 did not specifically target or internalize into normal mammary cells (Figure S2), normal prostate epithelial cells (Figure S3), or CD19+ B cells or CD3+ T cells from either unstimulated or stimulated bone marrow (Figure S4). Thus, E3 demonstrates selective nanomolar targeting to diverse cancer cell types, including cancers from six different tissues of origin, various subtypes of cancer within the different tissue types and cancer cells with differing receptor expression levels. 

### 2.2. E3 Aptamer Targeting to Human PDX-Derived Cell Lines

Although human tumor-derived cell lines are the most broadly used tissue culture models for cancer studies and remain extremely important and useful models, they do not always fully recapitulate the complexity of human tumors (reviewed in [26]) and often experience major changes in biology compared to their tumor of origin [27]. Due to these limitations, patient-derived tumor xenograft (PDX) models have risen to prominence as more accurate representations of actual human tumors (reviewed in [28,29]). PDX models are established from fresh human tumor pieces that are implanted into mice and subsequently grow into tumors in the mice. Cell suspensions can also be made from the tumors propagated in mice, creating PDX-derived cell lines. Therefore, to determine whether the E3 aptamer also targets more clinically relevant PDX-derived cell lines, we profiled E3 binding to 7 different PDX-derived cell lines. The panel of PDX-derived cell lines included 4 different colorectal cancer lines, a cholangiocarcinoma line, an osteosarcoma line and a renal cancer line. 

**Figure 2:**
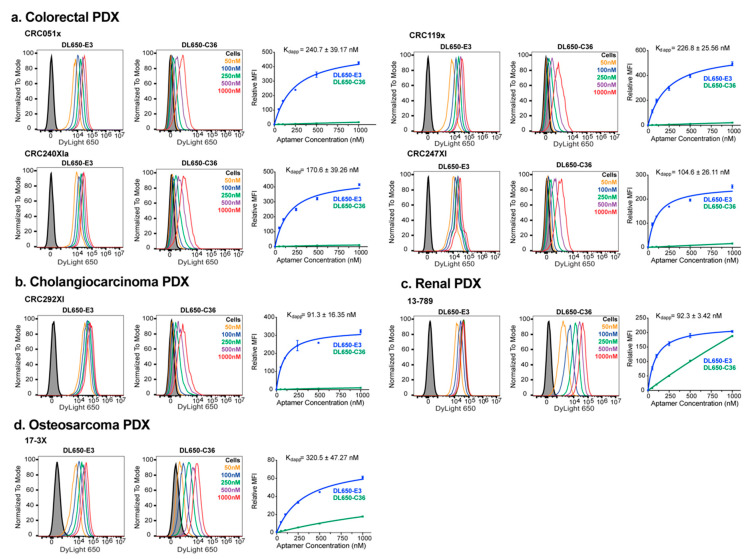
The E3 aptamer targets broadly across different PDX-derived cancer cells. Cancer cells were incubated with DL650-E3 aptamer or DL650-C36 control aptamer and analyzed, as described in Figure 1. The binding curves are the combination of three independent experiments, with the median fluorescent intensity (MFI) of the aptamer-treated samples normalized to the median fluorescent intensity of the cells alone signal. Flow cytometry analysis of E3 targeting to (**a**) the colorectal PDX-derived cell lines CRC051x, CRC119x, CRC240XIa, and CRC247XI; (**b**) the cholangiocarcinoma PDX-derived cell line CRC292XI; (**c**) the renal cancer cell line 13-789; and (**d**) the osteosarcoma cancer cell line 17-3X.

Increasing concentrations of DL650-E3 were incubated with each PDX-cell line and aptamer targeting quantified via flow cytometry. As with the tumor-derived cell lines, E3 targeted each PDX-derived cell line tested, specifically binding the cancer cells compared to the control C36 aptamer (Figure 2). E3 targeted the 4 colorectal PDX-derived cell lines with nanomolar affinity as nonlinear fits of the relative median fluorescence vs. aptamer concentration gave K_d_s ranging from 105—241 nM (Figure 2a). Notably, the CRC119x colorectal cancer cells have been thoroughly characterized and shown to accurately reflect the corresponding patient tumor, maintaining salient histology and gene expression patterns [30]. Of the PDX-derived cell lines tested, E3 exhibited the highest affinity binding for the cholangiocarcinoma cell line (K_d_ = 91.3 nM) (Figure 2b). The renal PDX cell line, 13-789, bound E3 with an affinity similar to that of the cholangiocarcinoma PDX line (Figure 2c). However, unlike the other tumor-derived or PDX-derived cell lines, the 13-789 cells displayed particularly high levels of background binding, as evidenced by the higher level of nonspecific, control aptamer C36 binding to the cells; this nonspecific binding occurred despite the inclusion of salmon sperm DNA as a blocking agent to prevent nonspecific oligonucleotide interactions. The osteosarcoma cell line, 17-3X, had the lowest affinity binding for E3 (K_d_ = 320 nM) of the PDX-derived cell lines tested (Figure 2d) but still bound the aptamer better than the non-PDX U-118MG glioblastoma cell line. Thus, the E3 aptamer maintains high-affinity targeting to PDX-derived cell lines that more closely model actual patient tumors. 

### 2.3. Confocal Microscopy Verifies E3 Internalization into PDX-Derived Colorectal Cancer Cells

**Figure 3:**
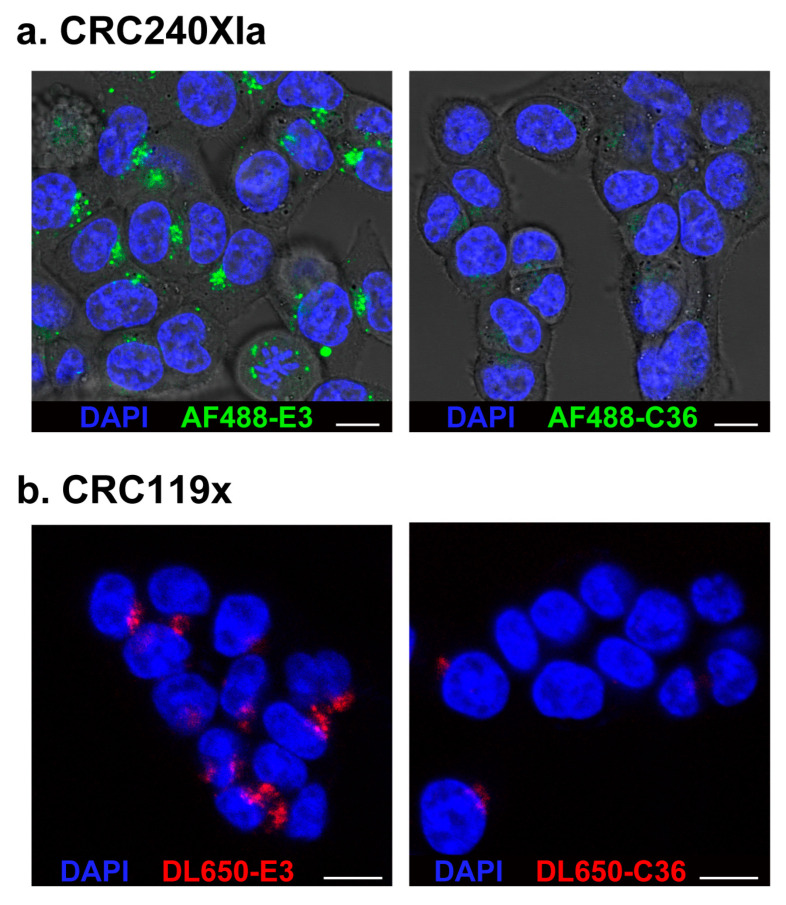
Confocal microscopy confirms E3 aptamer internalization into PDX-derived cancer cells. Cells were treated for 1 h with (**a**) 10 μM of AF488-E3 aptamer or AF488-C36 control aptamer or (**b**) 1 μM of DL650-E3 aptamer or DL650-C36 control aptamer. After washing, Hoechst 33342 was added to all samples to stain the nuclei. Cells were imaged on a Leica SP5 inverted confocal microscope. (White scale bars: 10 μm).

To determine if the E3 aptamer first binds and subsequently internalizes into PDX-derived cells, we synthesized E3 and conjugated it to either DL650 or to the dye Alexa Fluor 488 (AF488). Confocal microscopy revealed extensive E3 internalization into both the CRC240XIa and CRC119x colorectal PDX-derived cell lines, with punctate aptamer staining throughout the cytoplasm of both cell types, which contrasts to staining with the control aptamer C36 (Figure 3). Such cellular localization is consistent with the staining and accumulation of the E3 aptamer previously seen in prostate cancer cells [12], suggesting that E3 also enters PDX colorectal cancer cells via active-targeting receptor-mediated endocytosis, as in the case of prostate cancer cells. While internalization of dye-labeled E3 was visible by confocal microscopy at concentrations of 1 μM (Figure 3b, Figure S5), even a 10-fold higher concentration of dye-labeled control aptamer C36 did not internalize into the CRC240XIa colorectal cancer cell line (Figure 3a).

### 2.4. E3 MMAE and MMAF ApTDCs Inhibit Proliferation across a Range of Human Cancer Types

Based on the ability of both E3 MMAE and MMAF highly toxic drug conjugates to efficiently kill prostate cancer cells [12], we sought to determine whether these ApTDCs also show activity against the other human cancer cell lines that the E3 aptamer targets. Targeted delivery of MMAE and MMAF is particularly interesting to study as the auristatin derivates differ in membrane permeability. MMAE is membrane permeable in its unconjugated form, while MMAF is membrane impermeable. By conjugating the E3 aptamer to MMAE and MMAF using the same chemical linkers used for ADC versions of the drugs, both drugs remain membrane-impermeable unless they encounter the intracellular conditions required for drug release. This lability is achieved by using a valine-citrulline linker for MMAE, which ameliorates the membrane permeability of MMAE, preventing drug activity unless the drug internalizes into cells and reaches the lysosomes; the lysosomal protease cathepsin B cleaves the valine-citrulline portion of the linker, releasing free MMAE [21]. The free MMAE can then diffuse freely across the lysosomal membrane to reach the drug’s site of action in the cytoplasm, as both MMAE and MMAF function by binding tubulin and causing cell apoptosis [21,22]. As MMAF is inherently membrane-impermeable, its release from the lysosome is dependent upon conjugate degradation and eventual escape. 

To further examine binding vs. internalization of E3 into cells, we treated the CRC119x colorectal PDX-derived cells with DL650-E3 at either 4 °C or 37 °C. Cell internalization is prevented at 4 °C but can occur at the physiological temperature of 37 °C. As expected, E3 specifically targeted and bound the cells at 4 °C. Subsequent treatment with RNase to degrade aptamer bound to the cell surface resulted in a complete loss of the E3 signal, indicating that E3 is indeed on the cell surface and has not internalized into cells at 4 °C (Figure S6a). However, at 37 °C, DL650-E3 internalized into the cells and was not accessible to the RNase treatment (Figure S6b). Thus, E3 not only targets but also internalizes into PDX-derived cells. 

**Figure 4:**
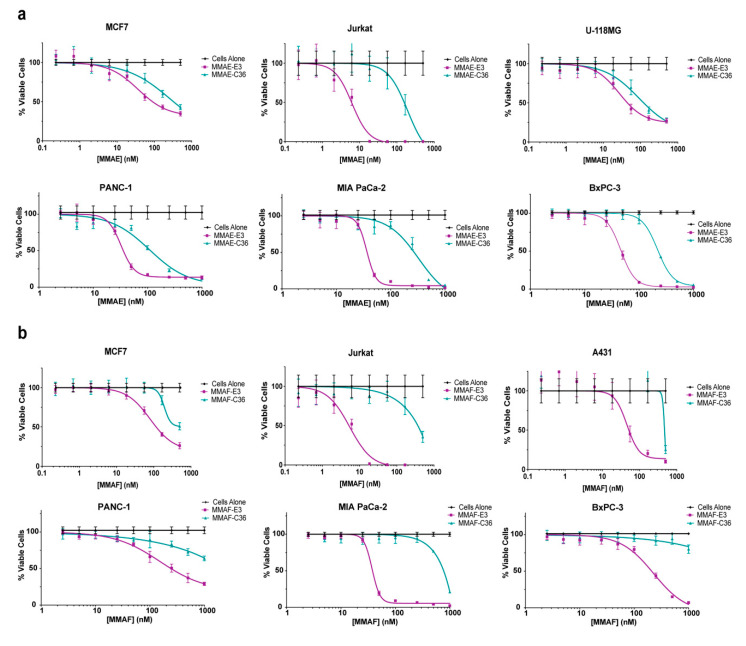

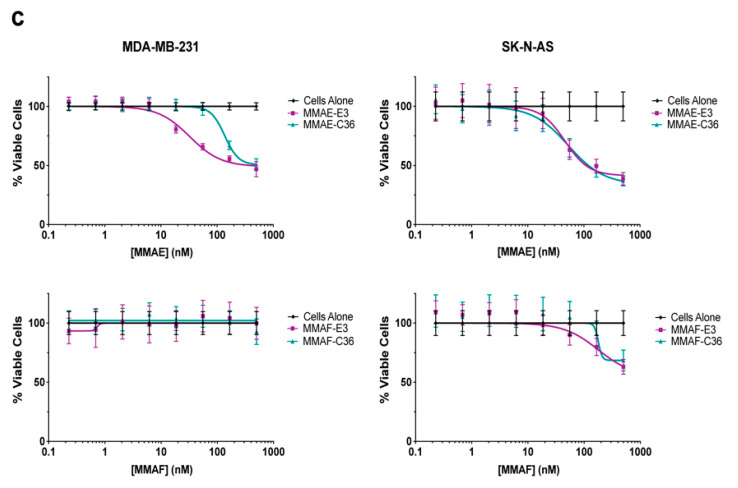
E3 MMAE and MMAF induce cell death in a broad number of cancer cells. (**a**) Viability of MCF7 breast, Jurkat blood and U-118MG brain cancer and PANC-1, MIA PaCa-2 and BxPC-3 pancreatic cancer cells treated with MMAF-E3 and MMAF-C36. (**b**) Viability of MCF7 breast, Jurkat blood and A431 skin cancer and PANC-1, MIA PaCa-2 and BxPC-3 pancreatic cancer cells treated with MMAF-E3 and MMAF-C36. (**c**) Viability of MDA-MB-231 breast and SK-N-AS brain cancer cells treated with MMAE-E3 and MMAE-C36 or with MMAF-E3 and MMAF-C36. The MMAE-C36 and MMAF-C36 data for the pancreatic cancer cells is from [13]. Cells were incubated with increasing concentrations of ApTDCs and viability determined at 144 h.

Consistent with the E3 aptamer’s ability to target a variety of different cancer cells, both the MMAE-E3 and MMAF-E3 conjugates caused cell killing across a range of different cancer cell types (Figure 4, Table S2). MMAE-E3 efficiently killed MCF7 breast cancer cells, Jurkat ALL cells, U-118MG glioblastoma cells, and the pancreatic cancer cell lines PANC-1, MIA PaCa-2, and BxPC-3 with IC_50_s in the nM range (Figure 4a). Jurkat cells were particularly sensitive to MMAE-E3, with an IC_50_ of only 6 nM. Although the MMAE-C36 conjugate did cause some cell death, this only occurred at the higher drug concentrations, likely due to extracellular degradation of the conjugate over the long time course of the experiment, leading to eventual internalization of free MMAE. Regardless, the MMAE-E3 conjugate was always at least ~3-fold more potent than the control C36 conjugate and, in the case of the Jurkat cells, was over 25 times as potent. 

Similarly, MMAF-E3 efficiently killed MCF7 breast cancer cells, Jurkat ALL cells, A431 skin epidermoid cancer cells, and the pancreatic cancer cell lines PANC-1, MIA PaCa-2 and BxPC-3 with IC_50_s in the nM range (Figure 4b). As with the MMAE conjugate, Jurkat cells were particularly sensitive to MMAF-E3, with an IC_50_ of only 5 nM. Interestingly, while MMAF-E3 had a similar IC_50_ value as MMAE-E3 on the MIA PaCa-2 cells, both the PANC-1 and BxPC3 cells were more sensitive to MMAE-E3 treatment than to MMAF-E3 treatment. For all of these cell lines, the control conjugate MMAF-C36 only induced cell death at the highest drug concentrations, never even reaching 50% cell death for the MCF7, PANC-1 or BxPC-3 cells. We previously reported the IC_50_s of free, unconjugated MMAE and MMAF on the pancreatic cancer cell lines PANC-1, MIA PaCa-2 and BxPC-3 [13]. MMAE-E3 was less efficient at killing the pancreatic cancer cells than free MMAE (Figure 4a and [13]), and MMAF-E3 was also less efficient at killing PANC-1 cells than free MMAF (Figure 4b and [13]). Interestingly, MMAF-E3 killed both MIA PaCa-2 and BxPC-3 cells more efficiently than free MMAF (free MMAE IC_50_ = 290 nM on both cell lines), with IC_50_s of 38 and 210 nM, respectively (Figure 4b and [13]). While these data demonstrate that E3 can improve drug targeting compared to unconjugated, free drug, MMAE and MMAF are too toxic to be used alone in the clinic [21,22]. Thus, conjugation to a targeting ligand such as E3 is necessary for clinical development irrespective of whether the targeting ligand improves drug efficacy. 

Although most cell lines are sensitive to at least one of the E3 drug conjugates, some cell lines are less sensitive than others, and the conjugates may not induce specific death in all cancer cell lines. 

For example, the MDA-MB-231 cells appear less sensitive to MMAE-E3 and MMAF-E3 (Figure 4c). While MMAE-E3 does kill the MDA-MB-231 cells more effectively compared to the control C36 conjugate, the IC_50_ of MMAE-E3 is much higher (~400 nM) in this cell line compared to most of the other cell lines tested. Additionally, MMAE-E3 does not appear to kill much more than 50% of the cell population. Likewise, the MMAF-E3 conjugate did not induce any cell death in the MDA-MB-231 cells. Interestingly, an ADC linked to MMAE using the same valine-citrulline linker has previously been shown to regress MDA-MB-231 tumor growth in mice, indicating that the cells are fully sensitive to the drug [31]. Thus, the MMAE and MMAF may not reach their site of action in the cytosol and may instead be trapped in an intracellular compartment such as an endosome or lysosome. Similarly, the MMAE-E3 and MMAF-E3 conjugates did not induce specific cell death in the SK-N-AS neuroblastoma cell line, with both targeted constructs giving similar cell viability profiles to the control, C36 conjugates (Figure 4c). The E3 and control C36 conjugates only induced cell death at high drug concentrations, similar to the cell death profiles of the C36 conjugates on the other cell lines tested, suggesting that the conjugates may also be trapped inside intracellular compartments in this cell line. Hence, the drug may only release into the cytosol at high concentrations, overriding any active drug targeting by E3. Thus, while the E3 MMAE and MMAF conjugates target and kill a variety of different cancer cell lines, E3 specificity for a cell line does not guarantee efficient drug-induced cell killing.

### 2.5. E3 MMAE and MMAF ApTDCs Inhibit Proliferation of Human PDX-Derived Cancer Cells

**Figure 5:**
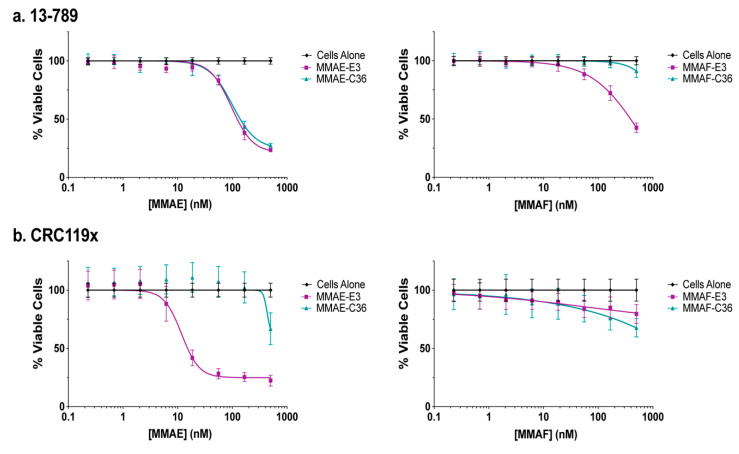
E3 MMAE and MMAF induce cell death in PDX-derived cancer cells. (**a**) Viability of 13-789 renal cancer PDX-derived cells and (**b**) CRC119x colorectal cancer PDX-derived cells. Cells were incubated with increasing concentrations of ApTDCs, and viability was determined at 144 h.

To determine whether the E3 MMAE and MMAF conjugates also show activity against more clinically relevant cancer cell lines, we tested the ability of the conjugates to induce cell death in PDX-derived cell lines. While the renal cancer cell line 13-789 was not more sensitive to MMAE-E3 than to the control MMAE-C36 conjugate, MMAF-E3 engendered 13-789 cell death more effectively than the MMAF control conjugate (Figure 5a, Table S2). This MMAF-E3 specificity occurred despite the high levels of nonspecific aptamer binding to these cells (Figure 2c), verifying that E3 does actively target the cells. Thus, the indistinguishable effects of MMAE-E3 and MMAE-C36 against these cells may be more a product of drug release or localization, with MMAE-E3 trapped in the endosomes or with an extracellular breakdown of the control conjugate allowing for free MMAE accumulation in cells. 

As expected, based upon E3 targeting and internalization into the CRC119x PDX-derived colorectal cancer cells, these cells were sensitive to MMAE-E3 drug targeting with an IC_50_ of 16 nM and very little nonspecific cell death caused by the control C36 conjugate (Figure 5b, Table S2). This result is particularly notable, given that CRC119x cells have been shown to closely model the parental patient tumor [30]. By contrast the MMAF-E3 conjugate did not specifically induce CRC119x cell death. As MMAF activity relies on passive breakdown and release from endosomes, instead of an active cathepsin-B dependent mechanism as with the MMAE conjugate, it is probable that the MMAF-E3 conjugate is stuck in intracellular compartments and thus the drug is not able to reach the cytosol. Given that both the MMAE-E3 and MMAF-E3 conjugates are active against different PDX-derived cancer cell lines, it is clear that E3 not only targets and internalizes into clinically relevant cell lines but can also efficiently deliver highly toxic drugs into such cells, inducing specific cell death.

### 2.6. The E3 Aptamer Targets PDX Colorectal Tumors In Vivo

**Figure 6:**
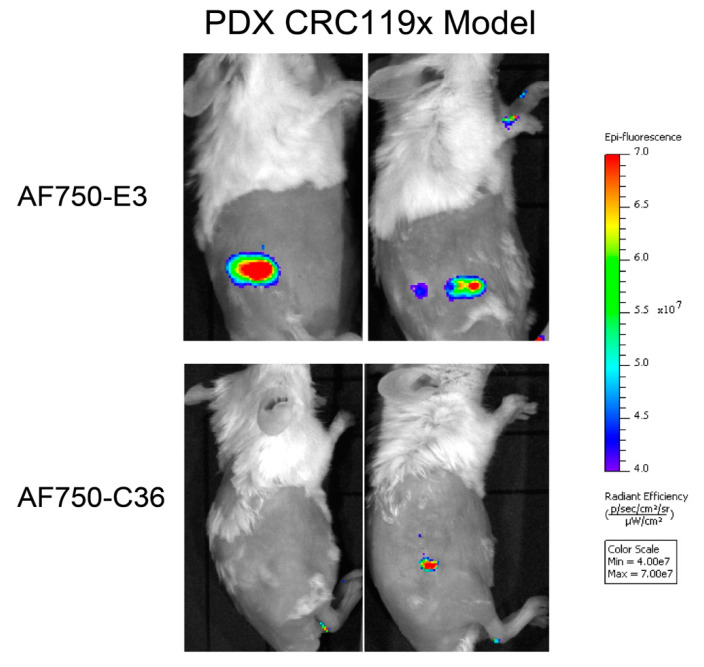
The E3 aptamer targets PDX tumors in vivo. PDX CRC119x colorectal tumors were established in the right flank of NOD/ScID mice. Tumor-bearing mice were injected via tail vein with 2 nmol of AF750-E3 (*n* = 3) or of control AF750-C36 (*n* = 2) and imaged for NIR fluorescence. Shown are representative images from 48 h post-aptamer injection.

Importantly, E3 targeting to clinically relevant PDX lines is maintained in PDX mouse models. Alexa Fluor 750 (AF750)-labeled E3 localizes to PDX colorectal CRC119x tumors in vivo, as can be visualized by near-infrared (NIR) imaging of tumor-bearing mice i.v. injected with the aptamer. As shown in Figure 6, AF750-E3 targets and accumulates in PDX tumors, while the control aptamer AF750-C36 does not. Thus, the E3 aptamer maintains targeting and aptamer function in the context of colorectal cancer that recapitulates the patient tumor.

## 3. Discussion

The clinical development of ADCs now represents one of the fastest-growing fields of cancer therapeutics (reviewed in [4,5]), with 5 ADCs gaining FDA approval since June of 2019 alone [6,7,8,9,10]. These therapeutics succeed by targeting and delivering highly toxic chemotherapy more directly to tumors, helping to prevent unwanted drug accumulation and toxicity in normal tissue. However, antibody development is an extensive process requiring not only antibody humanization but also difficult chemical conjugation, resulting in a heterogeneous drug product. Thus aptamers are emerging as ligands with an antibody-like affinity that can be used in place of antibodies to create targeted drug constructs. As aptamers are easily amenable to chemical synthesis and modification, they are chemical products and do not require the extensive optimization, such as humanization, that is required for biological drug products. Additionally, the small size of aptamers should aid in tumor penetration, a significant concern for ADCs, as studies have shown that less than 0.1% of an antibody is often even able to reach the tumor (reviewed in [32]).

Only a few reports have appeared of aptamer conjugation to highly toxic agents, including two reports of aptamer conjugation to biological toxins ([33,34]). More recently, our labs as well as the Rossi lab, have demonstrated that aptamers can be conjugated to highly toxic chemotherapeutics to generate ApTDCs [12,13,14]. Only one of these ApTDCs, the E3 aptamer MMAF conjugate, has been tested in vivo [12]. E3 was selected via positive-negative Cell-Internalization SELEX for internalization into prostate cancer and not normal prostate cells. ApTDCs formed by conjugating E3 to either MMAE or MMAF efficiently targeted and killed prostate cancer cells without affecting normal prostate cancer cells. Most significantly, AF750-E3 localized to prostate xenografts in mice and treatment with MMAF-E3 significantly inhibited prostate tumor growth and prolonged survival in mice. 

While E3 was selected for specificity to prostate cancer cells over normal prostate cells, we sought to determine whether E3 and E3 ApTDCs are solely selective for prostate cancer or whether they also target additional tumor types. Here, we demonstrate that the E3 aptamer targets across a broad range of human cancer types, showing an affinity for breast, pancreatic, lung, colorectal, cholangiocarcinoma, glioblastoma, neuroblastoma, leukemia, renal, and skin cancers. The E3 MMAE and MMAF drug conjugates also target and induce cell death across a range of these various cancer cell types. Most notably, E3 also targets and internalizes into PDX-derived cell lines that more closely reflect actual patient tumors than standard cancer cell lines. E3 targeting to PDX cell lines extends to the E3 drug conjugates, with both MMAE-E3 and MMAF-E3 efficiently inducing cell death in certain PDX cell lines. Additionally, E3 localizes to colorectal PDX tumors in mice, highlighting the clinical potential of the aptamer.

While the exact cellular target of E3 is still under investigation, there exist several possibilities for E3′s specific targeting to cancerous versus normal cells. E3 may be targeting a receptor that is restrictively expressed on cancer cells and not expressed on normal cells. However, it is more likely that E3 targets a receptor that is significantly overexpressed on cancer cells and only expressed at low levels on normal cells, such as the folate receptor [35]. Alternatively, E3 may target a receptor that is turned on during certain disease states such as cancer and only very rarely found in normal tissue, such as the integrin α_v_β_6_ [36]. Additionally, E3 could target a protein whose cellular localization changes in cancer cells, moving the protein from an intracellular compartment to the cell surface, such as happens for the nuclear protein nucleolin, which localizes to the cell surface of cancer cells [37].

While the E3 MMAE/F drug conjugates do not elicit efficient or specific cell death in every cancer cell line tested (Figure 4c), this result is not surprising given the extensive heterogeneity of cancer cells as well as the extensive number of steps necessary for aptamer-mediated targeting to deliver the drug to its site of action in the cytosol. The E3 conjugates appear to internalize into cells via active-targeting receptor-mediated endocytosis and subsequently localize into endosomes and lysosomes, as E3 enters prostate cancer cells via a clathrin-dependent pathway and colocalizes with LysoTracker [12]. Thus, the aptamer conjugate must not only deliver the drug into the cells but also degrade in the endosomes or lysosomes to allow the drug to escape out of these intracellular compartments. For the MMAE conjugates, this degradation is expected to occur via cleavage of the valine-citrulline drug linker by the endosomal enzyme cathepsin B. Once cleaved from the aptamer, the membrane-permeable MMAE drug should be able to diffuse across the endosomal membrane to reach the cytosol. As the MMAF conjugates are attached to the aptamer via a non-cleavable linker, they require not active, but rather passive, degradation in the endosome or lysosome. Additionally, as MMAF is not membrane-permeable, it cannot diffuse across the cell membrane and instead must rely on the disruption of the endosomes or lysosomes or on leakage out of those compartments. Only then can the MMAF drug also reach the cytosol. Thus, for cell types with low cathepsin B levels or with less “leaky” endosomal-lysosomal pathways, the auristatin drugs may not be the best choice of drug to couple to E3 to generate ApTDCs against a particular cancer. Given the large number of other highly toxic drugs conjugated to the ADCs currently in the clinic, any number of such drugs exist that can be conjugated to E3 to generate other ApTDCs that might prove efficacious against a particular cancer and yield a highly targeted precision medicine approach. Indeed, one major advantage to using an aptamer for drug delivery rather than an antibody is the ease of chemical conjugation. Due to the chemical nature of aptamers and ease of chemically modifying them, attaching a different type or class of drug to an aptamer is a straightforward process.

Another advantage of ApTDCs is the ease at which they can be controlled and quickly inactivated via antisense oligonucleotide “antidotes” [38,39,40,41,42,43]. Accordingly, an antidote against E3 was shown to prevent both E3 cell targeting and E3 MMAE and MMAF drug conjugate toxicity [12]. This trait is particularly useful in the context of targeted drug delivery as many antibody drugs have been shown to specifically accumulate in normal tissue as well as tumor tissue and to induce problematic toxicities in this normal tissue [44,45,46,47]. This accumulation is not an off-target effect but rather an effect of the antibody binding to its target when that target is found at a lower level in normal tissue. Thus, the ability to quickly inactivate E3 targeting serves as a promising control mechanism should the issue of toxicity in normal tissue arise.

## 4. Materials and Methods 

### 4.1. Cell Culture

Cells were obtained from the Duke University Cell Culture Facility (Durham, NC, USA) or derived from PDX models. All cells were grown at 37 °C with 5% CO_2_, and commercially available cell lines were cultured in media and supplements according to ATCC recommendations. The PDX-derived cells that begin with “CRC” were cultured in RPMI-1640 media (Invitrogen, Carlsbad, CA, USA) with 10% FBS (Sigma-Aldrich, St. Louis, MO, USA). The PDX-derived 13-789 and 17-3X cells were cultured in Dulbecco’s Modified Eagle Medium (Invitrogen, Carlsbad, CA, USA) with 10% FBS (Sigma-Aldrich, St. Louis, MO, USA). 

### 4.2. Aptamer Synthesis and Aptamer-Dye or Drug Conjugation

Synthesis of the aptamers and conjugation to dyes and drugs was performed as described previously [12]. Briefly, the E3 (GGC UUU CGG GCU UUC GGC AAC AUC AGC CCC UCA GCC) and C36 (GGC GUA GUG AUU AUG AAU CGU GUG CUA AUA CAC GCC) aptamers were synthesized using 2′-F-modified pyrimidines and 2′OH purines on an inverted dT CPG column by solid-phase synthesis. A 5′thiol C6 disulfide linker was added to the aptamer and reduced by incubating with 500 nM TCEP. The maleimide activated dyes, AF488, DL650, or AF750 (Invitrogen, Carlsbad, CA) or the auristatin derivates maleimide-caproyl-valine-citrulline-p-aminobenzylcarbamate-MMAE or maleimidocaproyl-MMAF (Levena Biopharma, San Diego, CA, USA) were added to the free thiol end of the aptamer. Conjugation efficiency was confirmed via analytical HPLC.

### 4.3. Flow Cytometry Analysis of Aptamer Binding 

Each cell line was plated in a 24 well plate at 90,000–100,000 cells/well and incubated at 37 °C, 5% CO_2_ for 2 days. Pancreatic cancer cells were instead plated at 50,000 cells/well in 96 well plates and incubated overnight. After removing the media, a 180 μL (for 24 well plates) or 90 μL (for 96 well plates) solution of 1.1 mg/mL salmon sperm DNA (MilliporeSigma, St. Louis, MO, USA) in complete media was added to each well for 1 h. DL650-aptamer solutions were prepared at 10x concentration in DPBS with Ca^2+^ and Mg^2+^ (ThermoFisher, Waltham, MA, USA) and folded at 65–70 °C for 5 min before renaturing at 4 °C for 5 min or RT for 15 min. 20μL (for 24 well plates) or 10 μL (for 96 well plates) of the aptamer solutions were added into appropriate wells in the plates and incubated for 1hr. Cells were then washed 2x with DPBS before adding 0.25% Trypsin (Invitrogen). Trypsin was quenched with complete media and cells transferred to a 96-well round bottom plate for subsequent centrifugation to generate cell pellets. Each pellet was resuspended in 100 μL of DPBS + 1% BSA and analyzed on a BD FACSCanto™ II (BD Biosciences, San Jose, CA, USA) or an iCyt Eclipse EC800 (Sony, New York, NY, USA). All samples were done in triplicate. 

### 4.4. Cell Viability Assays

Cells were seeded at 2000 cells/well or 4000 cells/well (pancreatic cancer cells only) in 96 well plates in a volume of 50 μL complete media. After 24 h, 40 μL of a 2.5 mg/mL salmon sperm DNA solution (MilliporeSigma, St. Louis, MO, USA) was added to each well and incubated on the cells for 1 h. Aptamer-drug conjugate solutions were prepared at 10x concentration (2.29–1000 nM based on drug concentration) in DPBS with Ca^2+^ and Mg^2+^ by folding at 65–70 °C for 5 min and renaturing at 4 °C for 5 min or RT for 15 min. 10 μL of the aptamer-drug solutions were then added to appropriate wells in quadruplicate, and cells maintained at 37 °C and 5% CO_2_. After 96 h, the media was removed from the pancreatic cancer cells and replaced with fresh media. At 144 h, viability was determined for the pancreatic cancer cell lines using AlamarBlue (Invitrogen, Carlsbad, CA, USA) and for all other cell lines using the CellTiter-Glo Luminescent Cell Viability Assay (Promega, Fitchburg, WI, USA). Data shown are the averages of 3–4 independent experiments.

### 4.5. Confocal Microscopy 

CRC240XIa or CRC119X cells were plated at 50,000–100,000 cells per dish into poly(D-lysine) coated glass-bottom culture dishes (No. 1.5 coverglass, MatTek Corporation, Ashland, MA, USA) and incubated overnight at 37 °C and 5% CO_2_. Cells were then incubated with 1 mg/mL salmon sperm DNA (ssDNA) solution (MilliporeSigma, St. Louis, MO, USA) in complete media for 1 h. During this incubation, aptamer-dye solutions were folded in DPBS with Ca^2+^ and Mg^2+^ at 65 °C for 5 min followed by 4 °C for 5 min. Cells were then treated for 1 h with 1 or 10 μM of AF488 or DL650-labeled E3 or C36 in complete media with 1 mg/mL ssDNA. All cells were then washed 3× with complete media before the addition of 1 mL complete media. 2 drops of NucBlue^®^ Live ReadyProbes^®^ Reagent (ThermoFisher, Waltham, MA, USA) were then added to each dish and incubated at room temperature for 20 min. Cells were imaged on a Lecia SP5 inverted confocal microscope (Leica Microsystems Inc., Buffalo Grove, IL, USA).

### 4.6. Establishment of PDX Mouse Models

The animal protocol used in this work was evaluated and approved by the Duke University Institutional Animal Care and Use Committee (Protocol: A194-12-07). Mice bearing CRC119x PDX tumors were established as previously described [30]. 

### 4.7. In Vivo Near Infrared Imaging

Mice bearing CRC119x tumors in the right flank were injected via tail vein with 2 nmol AF750-E3 or AF750-C36 and imaged at different time points up to 48 h post-aptamer injection on an IVIS Lumina XR (PerkinElmer, Waltham, MA, USA).

## 5. Conclusions

Thus, E3 appears to serve as a general cancer targeting ligand, capable of specifically targeting every cancer type tested to date. The activity of E3 drug conjugates against different cancer types coupled with the ability of the E3 aptamer to target PDX tumors in mice suggests that the E3 aptamer represents a general aptamer targeting platform for the delivery of highly toxic drugs to different cancers.

## 6. Patents

b3 bio, Inc. (A.P.B.) and Duke University (B.P.G. and B.A.S.) have submitted patent applications.

## Supplementary Materials

The following are available online at https://www.mdpi.com/2072-6694/12/11/3217/s1, Table S1: Description of cancer cells, Table S2: IC_50_ values of E3 and control aptamer, C36, drug conjugates on different cancer cell lines and PDX-derived cell lines, Figure S1: The E3 aptamer targets a broad range of cancer types, Figure S2: The E3 aptamer internalizes into breast cancer, but not normal mammary epithelial cells, Figure S3: The E3 aptamer specifically targets and internalizes into prostate cancer but not normal prostate epithelial cells, Figure S4: The E3 aptamer does not specifically internalize into normal bone marrow T or B cells, Figure S5: Confocal microscopy confirms E3 aptamer internalization into CRC240XIa PDX-derived cells, Figure S6: The E3 aptamer specifically binds and internalizes into CRC119x PDX-derived cells.

## References

[1] American Cancer Society (2019). Cancer Facts & Figures 2019.

[2] DeVita V.T., Chu E. (2008). A history of cancer chemotherapy. Cancer Res..

[3] Strebhardt K., Ullrich A. (2008). Paul Ehrlich’s magic bullet concept: 100 years of progress. Nat. Rev. Cancer.

[4] Khongorzul P., Ling C.J., Khan F.U., Ihsan A.U., Zhang J. (2020). Antibody-Drug Conjugates: A Comprehensive Review. Mol. Cancer Res..

[5] Lambert J.M., Berkenblit A. (2018). Antibody–Drug Conjugates for Cancer Treatment. Annu. Rev. Med..

[6] U.S. Food and Drug Administration (2019). FDA Approves Polatuzumab Vedotin-Piiq for Diffuse Large B-Cell Lymphoma. https://www.fda.gov/drugs/resources-information-approved-drugs/fda-approves-polatuzumab-vedotin-piiq-diffuse-large-b-cell-lymphoma.

[7] U.S. Food and Drug Administration (2019). FDA Approves New Type of Therapy to Treat Advanced Urothelial Cancer. https://www.fda.gov/news-events/press-announcements/fda-approves-new-type-therapy-treat-advanced-urothelial-cancer.

[8] U.S. Food and Drug Administration (2019). FDA Approves Fam-Trastuzumab Deruxtecan-Nxki for Unresectable or Metastatic HER2-Positive Breast Cancer. https://www.fda.gov/drugs/resources-information-approved-drugs/fda-approves-fam-trastuzumab-deruxtecan-nxki-unresectable-or-metastatic-her2-positive-breast-cancer.

[9] U.S. Food and Drug Administration (2020). FDA Grants Accelerated Approval to Sacituzumab Govitecan-Hziy for Metastatic Triple Negative Breast Cancer. https://www.fda.gov/drugs/drug-approvals-and-databases/fda-grants-accelerated-approval-sacituzumab-govitecan-hziy-metastatic-triple-negative-breast-cancer.

[10] U.S. Food and Drug Administration (2020). FDA Granted Accelerated Approval to Belantamab Mafodotin-Blmf for Multiple Myeloma. https://www.fda.gov/drugs/drug-approvals-and-databases/fda-granted-accelerated-approval-belantamab-mafodotin-blmf-multiple-myeloma.

[11] Chari R.V.J. (1998). Targeted delivery of chemotherapeutics: Tumor-activated prodrug therapy. Adv. Drug Deliv. Rev..

[12] Powell Gray B., Kelly L., Ahrens D.P., Barry A.P., Kratschmer C., Levy M., Sullenger B.A. (2018). Tunable cytotoxic aptamer-drug conjugates for the treatment of prostate cancer. Proc. Natl. Acad. Sci. USA.

[13] Kratschmer C., Levy M. (2018). Targeted Delivery of Auristatin-Modified Toxins to Pancreatic Cancer Using Aptamers. Mol. Ther. Nucleic Acids.

[14] Yoon S., Huang K.W., Reebye V., Spalding D., Przytycka T.M., Wang Y., Swiderski P., Li L., Armstrong B., Reccia I. (2017). Aptamer-Drug Conjugates of Active Metabolites of Nucleoside Analogs and Cytotoxic Agents Inhibit Pancreatic Tumor Cell Growth. Mol. Ther. Nucleic Acids.

[15] Conrad R.C., Giver L., Tian Y., Ellington A.D. (1996). In vitro selection of nucleic acid aptamers that bind proteins. Methods Enzymol..

[16] Osborne S.E., Ellington A.D. (1997). Nucleic Acid Selection and the Challenge of Combinatorial Chemistry. Chem. Rev..

[17] Nimjee S.M., White R.R., Becker R.C., Sullenger B.A. (2017). Aptamers as Therapeutics. Annu. Rev. Pharmacol. Toxicol..

[18] Ellington A.D., Szostak J.W. (1990). In vitro selection of RNA molecules that bind specific ligands. Nature.

[19] Tuerk C., Gold L. (1990). Systematic evolution of ligands by exponential enrichment: RNA ligands to bacteriophage T4 DNA polymerase. Science.

[20] Yan A., Levy M. (2014). Cell internalization SELEX: In vitro selection for molecules that internalize into cells. Methods Mol. Biol..

[21] Doronina S.O., Toki B.E., Torgov M.Y., Mendelsohn B.A., Cerveny C.G., Chace D.F., DeBlanc R.L., Gearing R.P., Bovee T.D., Siegall C.B. (2003). Development of potent monoclonal antibody auristatin conjugates for cancer therapy. Nat. Biotechnol..

[22] Doronina S.O., Mendelsohn B.A., Bovee T.D., Cerveny C.G., Alley S.C., Meyer D.L., Oflazoglu E., Toki B.E., Sanderson R.J., Zabinski R.F. (2006). Enhanced activity of monomethylauristatin F through monoclonal antibody delivery: Effects of linker technology on efficacy and toxicity. Bioconjug. Chem..

[23] Wilner S.E., Wengerter B., Maier K., de Lourdes Borba Magalhães M., Del Amo D.S., Pai S., Opazo F., Rizzoli S.O., Yan A., Levy M. (2012). An RNA alternative to human transferrin: A new tool for targeting human cells. Mol. Ther. Nucleic Acids.

[24] Dai X., Cheng H., Bai Z., Li J. (2017). Breast Cancer Cell Line Classification and Its Relevance with Breast Tumor Subtyping. J. Cancer.

[25] Adamska A., Domenichini A., Falasca M. (2017). Pancreatic Ductal Adenocarcinoma: Current and Evolving Therapies. Int. J. Mol. Sci..

[26] Gillet J.-P., Varma S., Gottesman M.M. (2013). The clinical relevance of cancer cell lines. J. Natl. Cancer Inst..

[27] Gillet J.-P., Calcagno A.M., Varma S., Marino M., Green L.J., Vora M.I., Patel C., Orina J.N., Eliseeva T.A., Singal V. (2011). Redefining the relevance of established cancer cell lines to the study of mechanisms of clinical anti-cancer drug resistance. Proc. Natl. Acad. Sci. USA.

[28] Yoshida G.J. (2020). Applications of patient-derived tumor xenograft models and tumor organoids. J. Hematol. Oncol..

[29] Hidalgo M., Amant F., Biankin A.V., Budinská E., Byrne A.T., Caldas C., Clarke R.B., de Jong S., Jonkers J., Mælandsmo G.M. (2014). Patient-derived xenograft models: An emerging platform for translational cancer research. Cancer Discov..

[30] Uronis J.M., Osada T., McCall S., Yang X.Y., Mantyh C., Morse M.A., Lyerly H.K., Clary B.M., Hsu D.S. (2012). Histological and Molecular Evaluation of Patient-Derived Colorectal Cancer Explants. PLoS ONE.

[31] Szot C., Saha S., Zhang X.M., Zhu Z., Hilton M.B., Morris K., Seaman S., Dunleavey J.M., Hsu K.-S., Yu G.-J. (2018). Tumor stroma-targeted antibody-drug conjugate triggers localized anticancer drug release. J. Clin. Investig..

[32] Christiansen J., Rajasekaran A.K. (2004). Biological impediments to monoclonal antibody–based cancer immunotherapy. Mol. Cancer Therapeutics.

[33] Kelly L., Kratschmer C., Maier K.E., Yan A.C., Levy M. (2016). Improved Synthesis and In Vitro Evaluation of an Aptamer Ribosomal Toxin Conjugate. Nucleic Acid Ther..

[34] Chu T.C., Marks J.W., Lavery L.A., Faulkner S., Rosenblum M.G., Ellington A.D., Levy M. (2006). Aptamer: Toxin conjugates that specifically target prostate tumor cells. Cancer Res..

[35] Weitman S.D., Lark R.H., Coney L.R., Fort D.W., Frasca V., Zurawski V.R., Kamen B.A. (1992). Distribution of the Folate Receptor GP38 in Normal and Malignant Cell Lines and Tissues. Cancer Res..

[36] Bandyopadhyay A., Raghavan S. (2009). Defining the role of integrin alphavbeta6 in cancer. Curr. Drug Targets.

[37] Hovanessian A.G., Soundaramourty C., Khoury D.E., Nondier I., Svab J., Krust B. (2010). Surface Expressed Nucleolin Is Constantly Induced in Tumor Cells to Mediate Calcium-Dependent Ligand Internalization. PLoS ONE.

[38] Rusconi C.P., Scardino E., Layzer J., Pitoc G.A., Ortel T.L., Monroe D., Sullenger B.A. (2002). RNA aptamers as reversible antagonists of coagulation factor IXa. Nature.

[39] Nimjee S.M., Keys J.R., Pitoc G.A., Quick G., Rusconi C.P., Sullenger B.A. (2006). A novel antidote-controlled anticoagulant reduces thrombin generation and inflammation and improves cardiac function in cardiopulmonary bypass surgery. Mol. Ther..

[40] Dyke C.K., Steinhubl S.R., Kleiman N.S., Cannon R.O., Aberle L.G., Lin M., Myles S.K., Melloni C., Harrington R.A., Alexander J.H. (2006). First-in-human experience of an antidote-controlled anticoagulant using RNA aptamer technology: A phase 1a pharmacodynamic evaluation of a drug-antidote pair for the controlled regulation of factor IXa activity. Circulation.

[41] Rusconi C.P., Roberts J.D., Pitoc G.A., Nimjee S.M., White R.R., Quick G., Scardino E., Fay W.P., Sullenger B.A. (2004). Antidote-mediated control of an anticoagulant aptamer in vivo. Nat. Biotechnol..

[42] Bompiani K.M., Monroe D.M., Church F.C., Sullenger B.A. (2012). A high affinity, antidote-controllable prothrombin and thrombin-binding RNA aptamer inhibits thrombin generation and thrombin activity. J Thromb. Haemost..

[43] Gunaratne R., Kumar S., Frederiksen J.W., Stayrook S., Lohrmann J.L., Perry K., Bompiani K.M., Chabata C.V., Thalji N.K., Ho M.D. (2018). Combination of aptamer and drug for reversible anticoagulation in cardiopulmonary bypass. Nat. Biotechnol..

[44] Lacouture M.E. (2006). Mechanisms of cutaneous toxicities to EGFR inhibitors. Nat. Rev. Cancer.

[45] Macdonald J.B., Macdonald B., Golitz L.E., LoRusso P., Sekulic A. (2015). Cutaneous adverse effects of targeted therapies: Part I: Inhibitors of the cellular membrane. J. Am. Acad. Dermatol..

[46] Onitilo A.A., Engel J.M., Stankowski R.V. (2014). Cardiovascular toxicity associated with adjuvant trastuzumab therapy: Prevalence, patient characteristics, and risk factors. Ther. Adv. Drug Saf..

[47] Liu S., Kurzrock R. (2014). Toxicity of targeted therapy: Implications for response and impact of genetic polymorphisms. Cancer Treat. Rev..

