# Supplementary material for: An Aptamer for Broad Cancer Targeting and Therapy
Source: Cancers (Basel). 2020 Oct 31;12(11):3217. doi: 10.3390/cancers12113217 (PMC7694147; doi:10.3390/cancers12113217)
Supplement: Supplementary file 1 [file cancers-12-03217-s001.pdf]

Supplementary Material

An Aptamer for Broad Cancer Targeting and Therapy

Bethany Powell Gray, Xirui Song, David S. Hsu, Christina Kratschmer, Matthew Levy, Ashley P. Barry and Bruce A. Sullenger

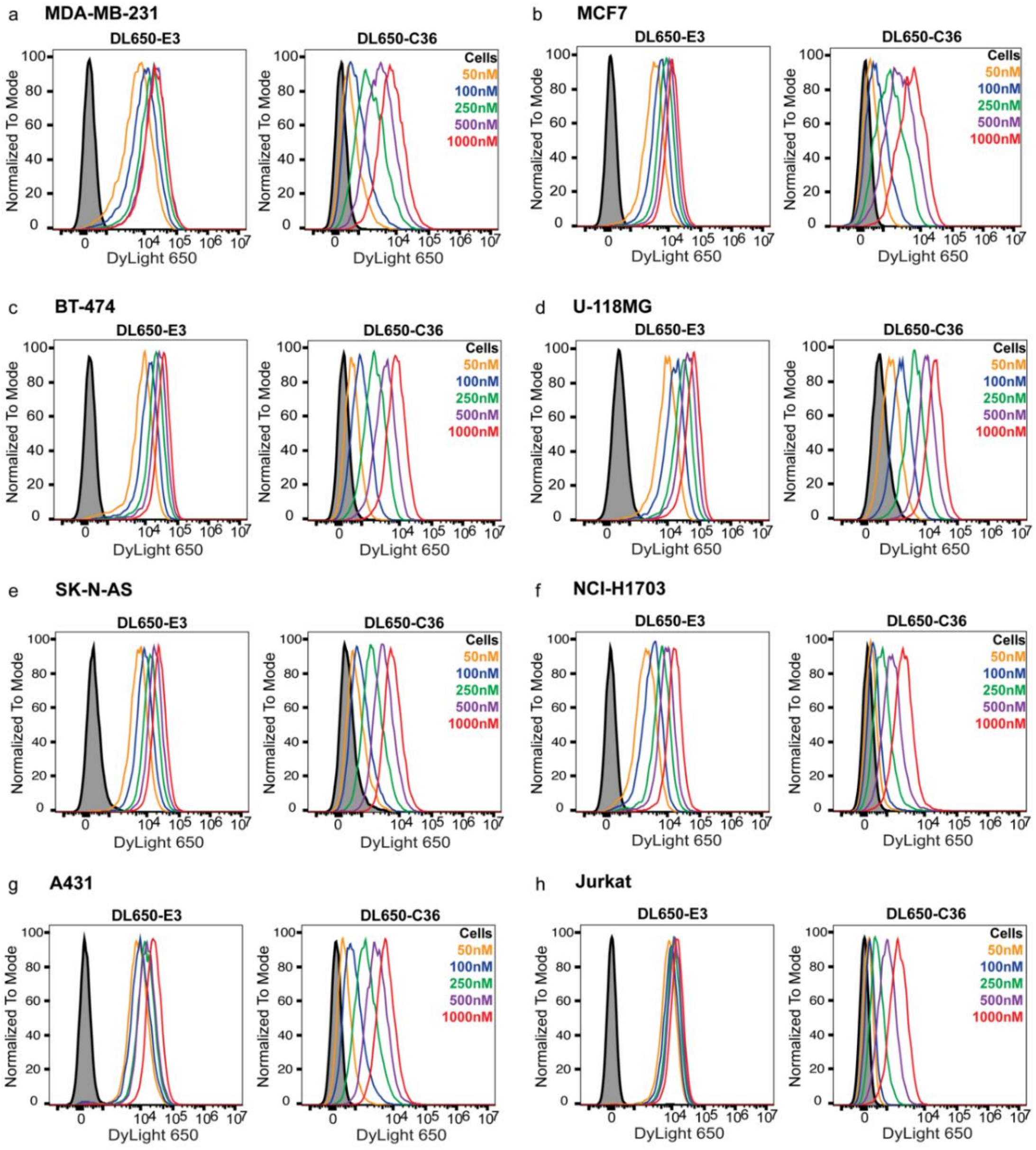

Figure S1. The E3 aptamer targets a broad range of cancer cell types. Cancer cells were incubated with increasing concentrations of DL650-E3 aptamer or DL650-C36 control aptamer for 1 h before washing cells and analyzing by flow cytometry. Flow cytometry analysis of E3 targeting the breast cancer cell lines (**a**) MDA-MB-231, (**b**) MCF7 and (**c**) BT-474; the brain cancer cells (**d**) U-118MG and (**e**) SK-N-AS; (**f**) the lung cancer cell line NCI-H1703; (**g**) the skin epidermoid cancer cell line A431; and (**h**) the Jurkat peripheral blood cancer cell line.

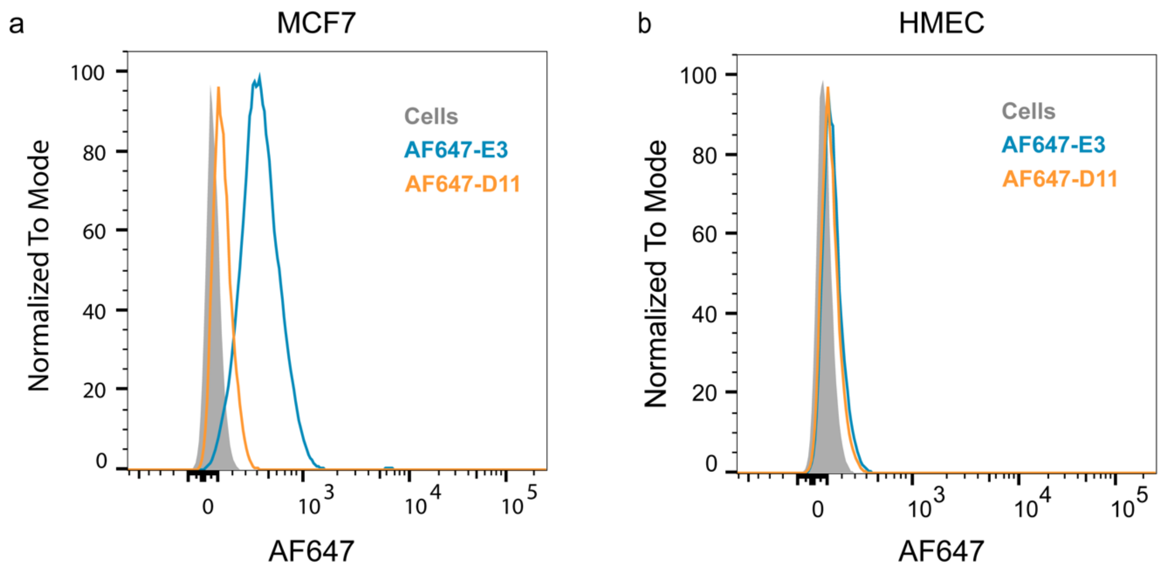

Figure S2. The E3 aptamer internalizes into breast cancer but not normal mammary epithelial cells. Cells were incubated with the E3 aptamer or D11 control aptamer annealed to an AF647-labeled reverse primer for 2 h before treating with Riboshredder to degrade cell surface bound aptamer. Cells were then washed and analyzed by flow cytometry. (**a**) Flow cytometry analysis shows E3 internalization into the breast cancer cell line MCF7. (**b**) Flow cytometry analysis shows that E3 does not internalize into HMEC normal mammary epithelial cells.

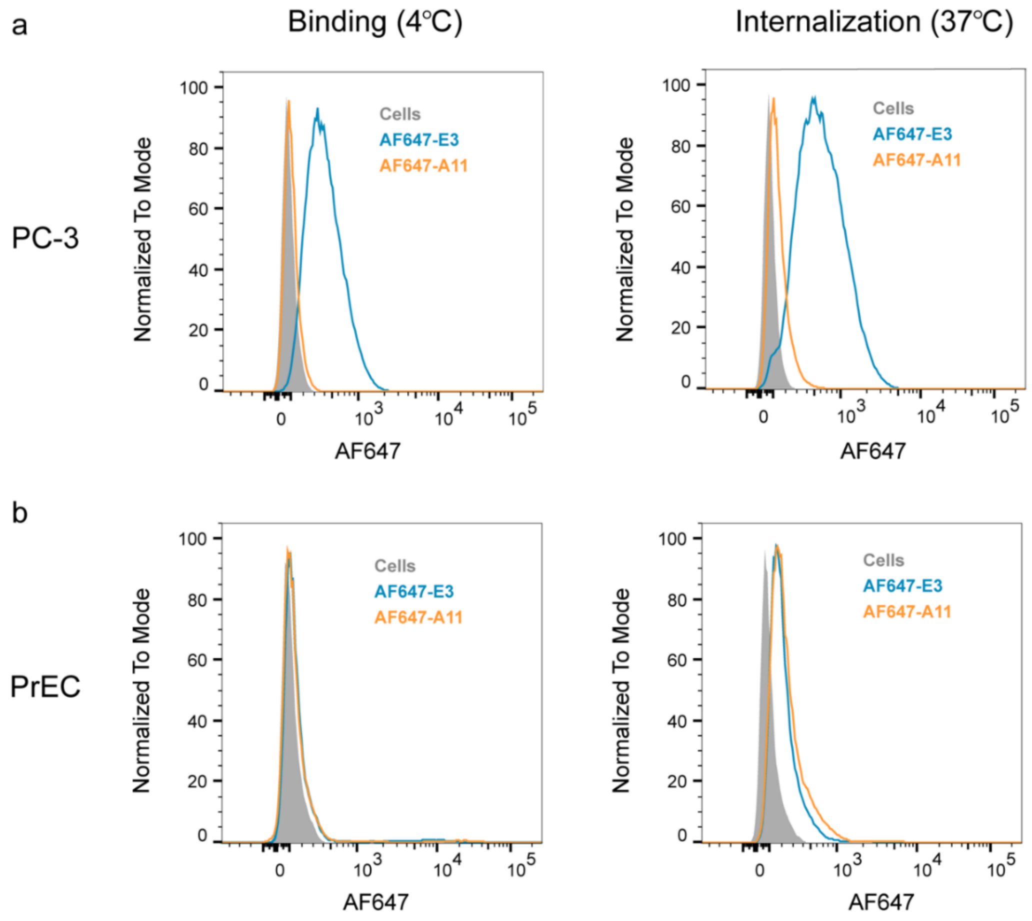

Figure S3. The E3 aptamer specifically targets and internalizes into prostate cancer but not normal prostate epithelial cells. Cells were incubated with the E3 aptamer or A11 control aptamer annealed to an AF647-labeled reverse primer. For binding studies, cells were treated with aptamer for 30 min

at 4 °C. For internalization studies, cells were treated with aptamer for 2 h at 37 °C and subsequently incubated with Riboshredder to degrade aptamer bound to the cell surface before washing cells and analyzing by flow cytometry. (**a**) Flow cytometry analysis shows that E3 both binds and internalizes into PC-3 prostate cancer cells. (**b**) Flow cytometry analysis shows that E3 does not bind PrEC normal prostate epithelial cells at 4 °C and only minimally internalizes into the cells at 37 °C in a nonspecific fashion, identical to that of a control, non-binding aptamer.

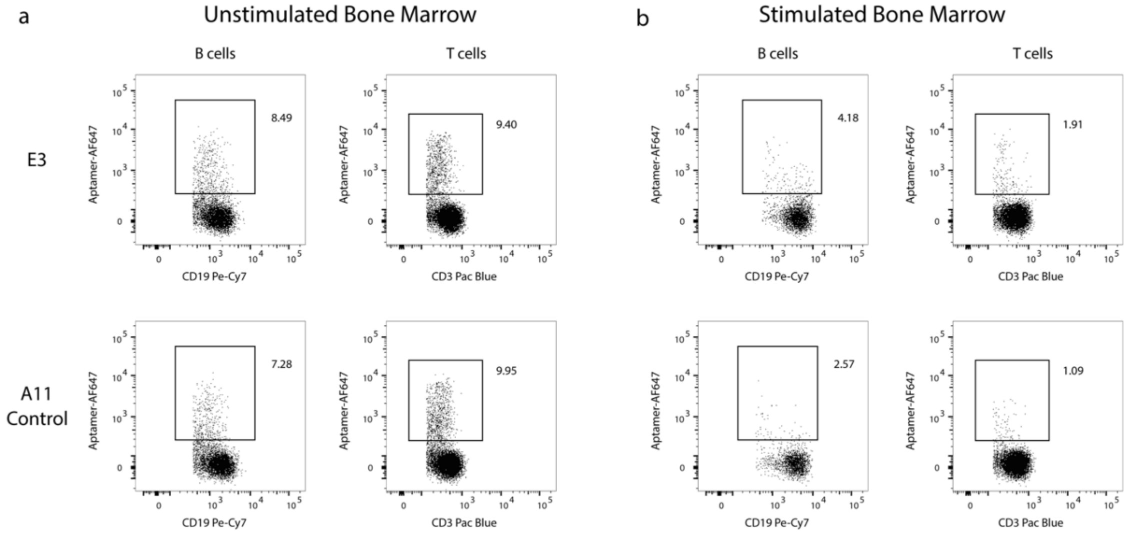

Figure S4. The E3 aptamer does not specifically internalize into normal bone marrow T or B cells. Cells were incubated with the E3 aptamer or A11 control aptamer annealed to an AF647-labeled reverse primer. Cells were treated with aptamer for 2 h at 37 °C and subsequently incubated with Riboshredder to degrade aptamer bound to the cell surface before washing cells and analyzing by flow cytometry. (**a**) Flow cytometry analysis shows that E3 does not specifically internalize into CD19+ B cells or CD3+ T cells from unstimulated bone marrow. (**b**) Flow cytometry analysis shows that E3 does not specifically internalize into CD19+ B cells or CD3+ T cells from stimulated bone marrow.

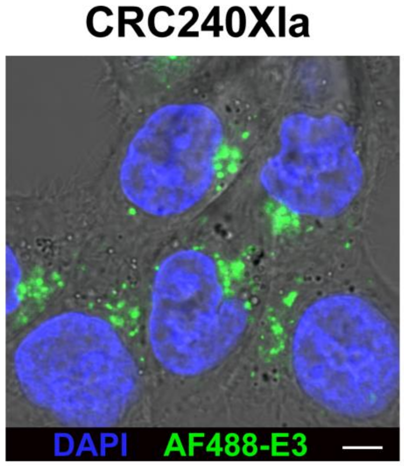

Figure S5. Confocal microscopy confirms E3 aptamer internalization into CRC240XIa, PDX-derived cells. Cells were treated for 1 h with 1 μM of AF488-E3 aptamer. After washing, Hoechst 33342 was added to stain the nuclei. Cells were imaged on a Leica SP5 inverted confocal microscope. (White scale bar: 5 μm)

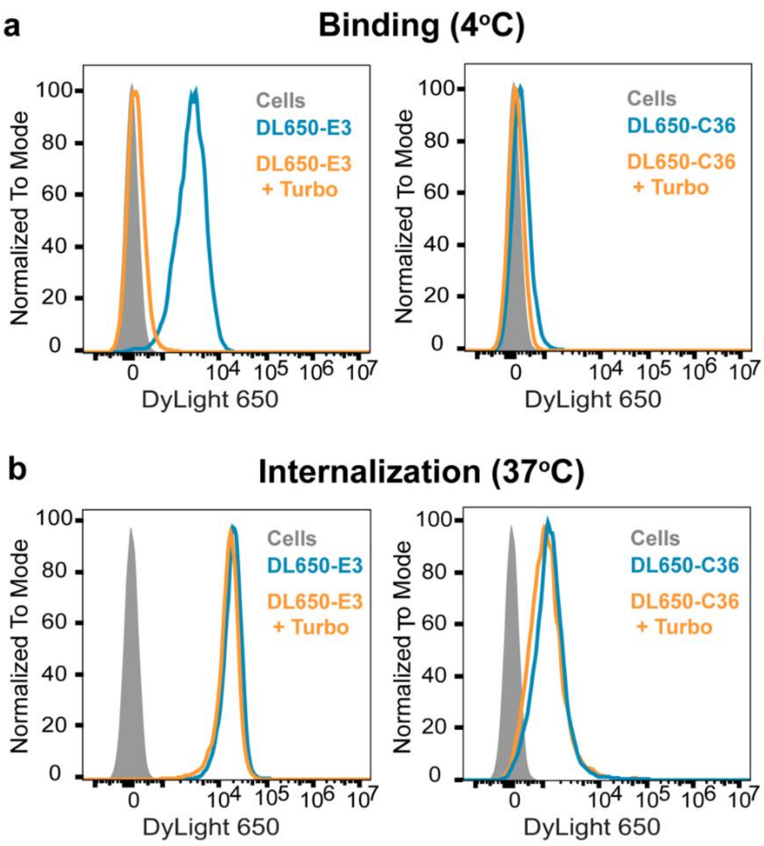

Figure S6. The E3 aptamer specifically binds and internalizes into CRC119x PDX-derived cells. Cells were incubated with DL650-E3 aptamer or DL650-C36 control aptamer for 1h at either 4 °C or 37 °C before washing cells and subsequently treating some cells with Turbonuclease to degrade aptamer bound to the cell surface. Cells were then washed and analyzed by flow cytometry. (**a**) Flow cytometry analysis after binding studies at 4 °C shows that E3, but not control aptamer C36, specifically binds to the surface of CRC119x cells. Treatment with Turbonuclease (Turbo) completely degrades cell surface bound aptamer, taking the E3 signal back down to background. (**b**) Flow cytometry analysis after internalization studies at 37 °C shows that E3 internalizes into CRC119x cells. Treatment with Turbonuclease (Turbo) does not alter the E3 signal as the aptamer has internalized and is not available for degradation. Control aptamer C36 only has minimal nonspecific internalization into the cells.

Table S1. Description of Cancer Cells.

Tissue of Origin	Cell Line	Type	Notable Features
Breast	MDA-MB-231	Adenocarcinoma	Triple negative
	MCF7	Invasive ductal carcinoma	ER+, PR+,
	BT-474	Invasive ductal carcinoma	ER+, PR+, HER2+
Pancreas	Panc-1	Pancreatic ductal adenocarcinoma	
	MIA PaCa-2	Pancreatic ductal adenocarcinoma	
	BxPC-3	Pancreatic ductal adenocarcinoma	
Brain	U-118MG	Glioblastoma	
	SK-N-AS	Neuroblastoma	
Lung	NCI-H1703	Squamous cell carcinoma	
Peripheral blood	Jurkat	Acute lymphoblastic leukemia	
Skin	A431	Epidermoid carcinoma	

Table S2. IC_{50} Values of E3 and control aptamer, C36, drug conjugates on different cancer cell lines and PDX-derived cell lines.

Cell Line	Drug Formulation	IC_{50} (nM) ± SE
U-118MG	MMAE-E3	44.5 ± 1.26

	MMAE-C36	121 ± 1.35
MCF7	MMAE-E3	89.0 ± 1.53
	MMAE-C36	325 ± 10.2
	MMAF-E3	116 ± 1.08
	MMAF-C36	>500
Jurkat	MMAE-E3	6.08 ± 1.16
	MMAE-C36	157 ± 1.12
	MMAF-E3	5.21 ± 1.24
	MMAF-C36	368
A431	MMAF-E3	51.7 ± 1.33
	MMAF-C36	471
PANC-1	MMAE-E3	35.7 ± 1.05
	MMAE-C36	113 ± 1.39
	MMAF-E3	244 ± 1.20
	MMAF-C36	>1000
MIA PaCa-2	MMAE-E3	36.9 ± 1.06
	MMAE-C36	271 ± 1.51
	MMAF-E3	38.0 ± 1.04
	MMAF-C36	771
BxPC-3	MMAE-E3	46.8 ± 1.04
	MMAE-C36	225 ± 1.03
	MMAF-E3	210 ± 1.24
	MMAF-C36	>1000
MDA-MB-231	MMAE-E3	399 ± 1.33
	MMAE-C36	>500
	MMAF-E3	>500
	MMAF-C36	>500
SK-N-AS	MMAE-E3	109 ± 1.15
	MMAE-C36	120 ± 1.24
	MMAF-E3	>500
	MMAF-C36	>500
13-789	MMAE-E3	123 ± 1.14
	MMAE-C36	139 ± 1.10
	MMAF-E3	391 ± 1.46
	MMAF-C36	>500
CRC119x	MMAE-E3	15.2 ± 1.11
	MMAE-C36	>500
	MMAF-E3	>500
	MMAF-C36	>500

>500 or >1000 indicates that the IC_{50} is greater than the maximum drug concentration tested.

Supplemental Methods

Flow Cytometry Analysis of Aptamer Targeting to Cancer Cells

Each cell line was plated in a 24 well plate at 90,000–100,000 cells/well and incubated at 37 °C, 5% CO_2 for 2 days. After removing the media, a 180 µL solution of 1.1 mg/mL salmon sperm DNA (MilliporeSigma, St. Louis, MO, USA) in complete media was added to each well for 1 h. DL650-aptamer solutions were prepared at 10x concentration in DPBS with Ca^{2+} and Mg^{2+} (ThermoFisher, Waltham, MA, USA) and folded at 65 °C for 5 min before renaturing at 4 °C for 5 min. 20 µL of the aptamer solutions were added into appropriate wells in the plates and incubated for 1hr. Cells were then washed 2x with DPBS before adding 0.25% Trypsin (Invitrogen). Trypsin was quenched with complete media and cells transferred to a 96-well round bottom plate for subsequent centrifugation to generate cell pellets. Each pellet was resuspended in 100 µL of DPBS + 1% BSA and analyzed on a BD FACSCanto™ II (BD Biosciences, San Jose, CA, USA). All samples were done in triplicate.

Flow Cytometry Analysis of Aptamer Binding at 4 °C or Internalization at 37 °C for Mammary, Prostate and Bone Marrow Cells

Full length E3 aptamer (GGGAGGACGAUGCGGUACUUUCGGGCUUUCGGCA ACAUCAGCCCCUCAGGACGCAAUUUCUCCUACUGGGAUAGGUGGAUUAU) or control non-binding aptamers D11 (GGGAGGACGAUGCGGUCCCCGGAUUUCGGAUACGAUC CCUCAUCCCUUGACCGCAAUUUCUCCUACUGGGAUAGGUGGAUUAU) or A11 were transcribed with 2′F pyrimidines using Y639F mutant T7 polymerase. A 22 nucleotide tail was added to the 3′ ends of the aptamers during transcription to anneal to an AF647-labeled reverse primer. Frozen bone marrow mononuclear cells (AllCells, Alameda, CA, USA) were thawed according to the company's instructions. Bone marrow medium (BMM) was prepared by adding 30% FBS and 10mg/mL BSA to Iscove's Modified Dulbecco's Medium (IMDM). Bone marrow cells were stimulated by resuspending at 2 x 10^7 cells/mL in BMM supplemented with 10 ng/mL IL-3, 50 ng/mL rhSCF, 10 ng/mL GM-CSF, 10 ng/mL G-CSF and 3 U/mL EPO and incubated at 37 °C and 5% CO_2 for 72 h. Unstimulated bone marrow cells were resuspended at 2 x 10^6 cells/mL in IMDM with 30% FBS and incubated overnight at 37 °C and 5% CO_2.

Normal or cancerous prostate or breast or bone marrow cells were resuspended in FACS buffer (PBS + 0.5% BSA) supplemented with 100 µg/mL yeast tRNA at 3 x 10^6 cells/mL. Aptamers were folded and annealed to the AF647-labeled reverse primer by heating for 5 min at 85 °C in DPBS with 5 mM MgCl2 before cooling to 37 °C for 10min and then placing at 4 °C.

100 µL of cells were incubated with 200 nM labeled aptamer for 30 min on ice (for binding only studies) or 2 h at 37 °C for internalization studies. At the end of the 37 °C incubation, the samples were washed in FACS buffer and then split in half for +/- RiboShredder™ (Epicentre, Chicago, IL, USA) to determine internalization. 140 µL of RiboShredder™ was added to 3.5 mL of PBS, and 200 µL of the mixture added to the + RiboShredder™ samples and incubated for 20 min at 37°C. The samples that were not treated with RiboShredder™ were incubated with PBS only for 20 min at 37°C. Cells were then washed twice with PBS. The normal and cancerous prostate and breast cells were then fixed in 150 µL of 15% formaldehyde. The bone marrow cells were subsequently stained with CD8-FITC, CD34-PE, CD14-PerCP, CD19-PE-Cy7, CD3e-AF405 (all from BD Biosciences, San Jose, CA, USA) and LIVE/DEAD™ Fixable Aqua Dead Cell Stain (Invitrogen, Carlsbad, CA, USA) for 20 min at RT before fixation in 150 µL of 15% formaldehyde. All cells were then analyzed by flow cytometry.

Flow Cytometry Analysis of Aptamer Binding at 4 °C or Internalization at 37 °C for CRC119x PDX-Derived Cells

CRC119x cells were placed in tubes at 100,000 cells/tube. A 180 µL solution of 1.1 mg/mL salmon sperm DNA (MilliporeSigma, St. Louis, MO, USA) in complete media was added to each tube and incubated for 1 h either on ice (for binding studies) or at 37 °C (for internalization studies). DL650-aptamer solutions were prepared at 2.5 µM (10x) concentration in DPBS with Ca^{2+} and Mg^{2+} (ThermoFisher, Waltham, MA, USA) and folded at 65 °C for 5 min and 4 °C for 5 min. 20 µL of the aptamer solutions were added into appropriate tubes and incubated either on ice (for binding studies) or at 37 °C (for internalization studies) for 1 h. Cells were then washed with Hank's Balanced Salt Solution (HBSS, MilliporeSigma, St. Louis, MO, USA) before adding 100 µL of 2,000 U Turbonuclease (MilliporeSigma, St. Louis, MO, USA) diluted in HBSS to appropriate samples to remove cell surface-bound aptamers. Cells that were not treated with Turbonuclease were resuspended in HBSS alone. The +/- Turbonuclease samples were then incubated at 37 °C for 45min. Cells were then washed 3x times with DPBS and resuspended in 100 µL of DPBS + 1% BSA. Cells were transferred into a 96-well round bottom plate for analysis on a BD FACSCanto™ II (BD Biosciences, San Jose, CA, USA).

Confocal Microscopy

CRC240XIa cells were plated at 50,000 cells per dish into poly(D-lysine) coated glass-bottom culture dishes (No. 1.5 coverglass, MatTek Corporation, Ashland, MA, USA) and incubated overnight at 37 °C and 5% CO_2. Cells were then incubated with 1 mg/mL salmon sperm DNA (ssDNA) solution (MilliporeSigma, St. Louis, MO, USA) in complete media for 1 h. During this incubation, aptamer-dye solutions were folded in DPBS with Ca^{2+} and Mg^{2+} at 65 °C for 5 min followed by 4 °C for 5 min. Cells were then treated for 1 hr with 1 μM of AF488-E3 in complete media with 1 mg/mL ssDNA. All cells were then washed 3x with complete media before the addition of 1 mL complete media. 2 drops of NucBlue® Live ReadyProbes® Reagent (ThermoFisher, Waltham, MA, USA) were then added to each dish and incubated at room temperature for 20 min. Cells were imaged on a Lecia SP5 inverted confocal microscope (Leica Microsystems Inc., Buffalo Grove, IL, USA).
